# An exploratory study of circulating cell-free mRNAs across colorectal cancer stages

**DOI:** 10.1016/j.isci.2026.116398

**Published:** 2026-06-17

**Authors:** Thibault Mazard, Marie Grosgeorges, Laura Veyrie, Simon Thezenas, Brice Pastor, Ekaterina Pisareva, Gérald Lossaint, Evelyne Lopez-Crapez, Marc Ychou, Alain R. Thierry, Corinne Prévostel, Philippe Blache

**Affiliations:** 1 IRCM, University Montpellier, ICM, INSERM, CNRS Montpellier, Campus Val d’Aurelle 208 avenue des Apothicaires, 34298, Montpellier cedex 5, France; 2 ICM, Institut du Cancer de Montpellier, Campus Val d’Aurelle 208 avenue des Apothicaires, 34298, Montpellier cedex 5, France

**Keywords:** oncology, diagnostics, medical biochemistry, cancer

## Abstract

This study evaluated circulating mRNAs (B2M, TIMP-1, CLU, SERPINE1, GAL3, and S100A9) as potential biomarkers for colorectal cancer (CRC). Plasma levels were measured in CRC patients (stages I–IV, *n* = 178) and healthy individuals (HIs, *n* = 171). B2M, TIMP-1, CLU, and S100A9 mRNA levels were significantly elevated in CRC patients vs. HIs. B2M differed only in stage IV, while TIMP-1, CLU, and S100A9 showed significance across all stages except I. A composite index, I-BTC, was developed using B2M, TIMP-1, and CLU via logistic regression. I-BTC improved discrimination between HIs and CRC patients, even at stage I (*p* = 0.001), with stronger significance in later stages (*p* ≤ 0.0001). These results validate analytical robustness of the method and highlight its diagnostic potential including for early-stage CRC through specific mRNAs combinations.

## Highlights

• B2M, TIMP-1, CLU, and S100A9 mRNA levels in CRC patient plasmas
• I-BTC index combines B2M, TIMP-1, CLU mRNA levels into a logistic regression model
• I-BTC index improves early-stage CRC detection, especially for stage I
• Plasma mRNA assay shows diagnostic potential for CRC, including early stages

## Graphical abstract

**Figure:**
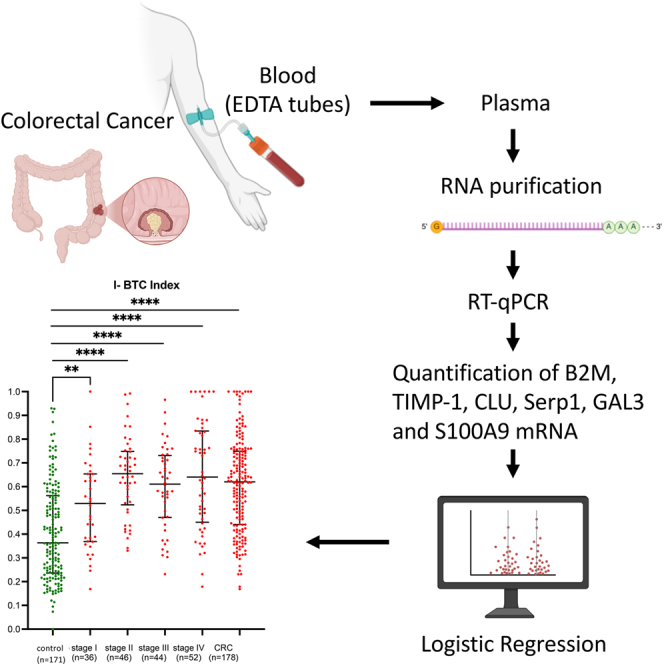

## Introduction

The risk of colorectal cancer (CRC) recurrence is closely linked to the stage of the disease at diagnosis.^1^^,^^2^ Indeed, early diagnosis significantly reduces the risk of death, as CRC is more effectively treated when it is detected at an early stage.^1^^,^^3^ Therefore, the current early detection methods for CRC, including fecal occult blood tests and fecal immunochemical tests (FITs), aim to reduce the incidence and mortality of CRC.^4^^,^^5^ However, their widespread adoption is limited by their low acceptability^6^^,^^7^^,^^8^ and insufficient specificity and sensitivity. As a result, three-quarters of CRC deaths occur in people who have not been screened,^9^ highlighting the need to develop innovative approaches for earlier diagnosis.^8^^,^^10^

To address this issue, we developed a reverse transcriptase quantitative polymerase chain reaction (RT-qPCR)-based method for analyzing circulating plasma mRNAs, referred to as *Plasma RNA for Cancer Check-up (RNA-2C)*.^11^ RNA2C has key advantages: its affordability, its rapid processing time, its minimal plasma volume requirement, and the use of an RNA extraction technique that complies with hospital health and safety standards, avoiding phenol-chloroform. We demonstrated that the mRNA levels of beta-2 microglobulin (B2M), tissue inhibitor of metalloproteinases 1 (TIMP-1), and clusterin (CLU) were significantly higher in patients with metastatic colorectal cancer (mCRC) than in healthy individuals (HI). This study^11^ was conducted via whole blood samples collected in Streck cell-free DNA BCT tubes,^12^ which are specifically designed for the analysis of circulating DNA in blood samples collected several days prior to plasma preparation.

According to recent studies, plasma mRNAs circulate within large and medium-sized protective particles,^13^ suggesting that preanalytical conditions, including centrifugation speed, blood collection methods, and plasma preparation time, may influence the final results. Therefore, one objective of the present study was to assess the applicability of our method to blood samples collected in EDTA tubes in patients with various stages of CRC. In addition, we sought to study the impact of surgery on circulating mRNA levels in patients with localized cancers.

## Results

### Comparison of Streck tubes and EDTA tubes for circulating mRNA analysis

**Figure 1:**
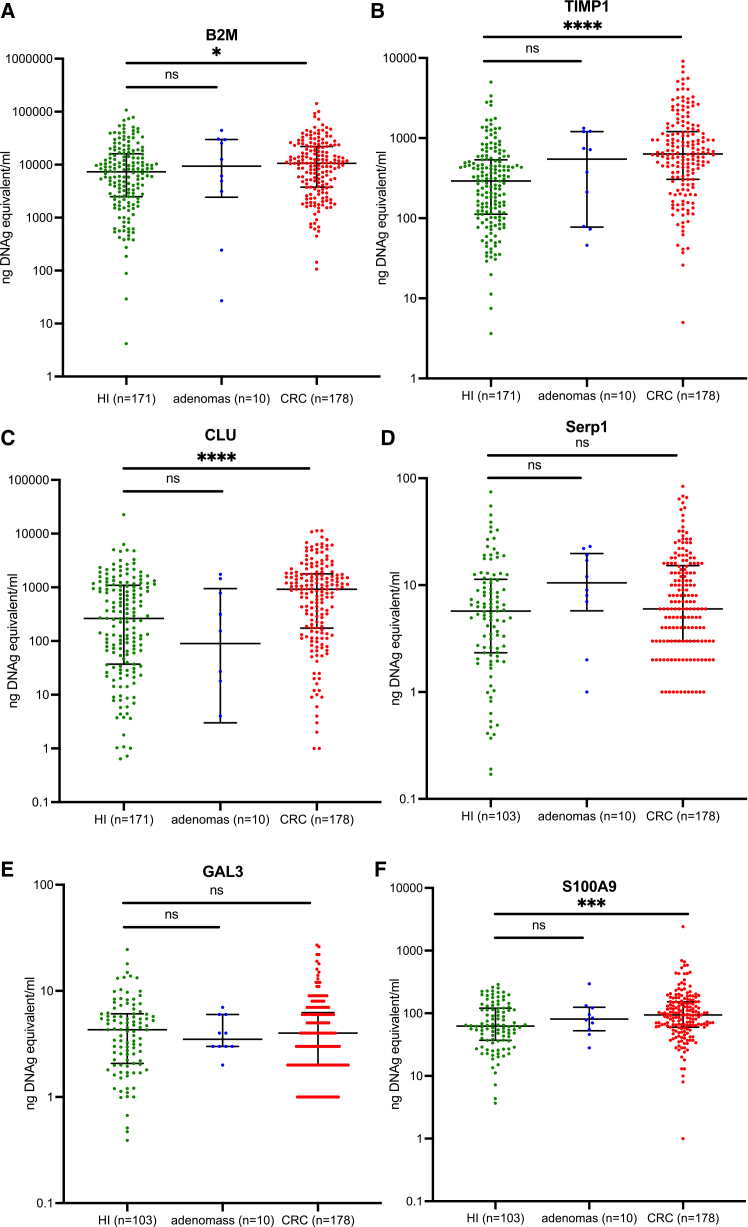
Circulating mRNA plasma in HI, patients with adenomas, and patients with CRC B2M (A), TIMP-1 (B), CLU (C), Serp1 (D), GAL3 (E), and S100A9 (F) mRNA plasma levels in HI, patients with advanced adenomas and patients with CRC. Medians are shown, and error bars indicate the interquartile range. All values and statistics are provided in Table 2. The results are expressed in ng human genomic DNA (DNAg) equivalent per mL. The non-parametric Mann-Whitney test was used, ∗*p* < 0.05; ∗∗*p* < 0.01; ∗∗∗*p* < 0.001; ∗∗∗∗*p* < 0.0001.

**Figure 2:**
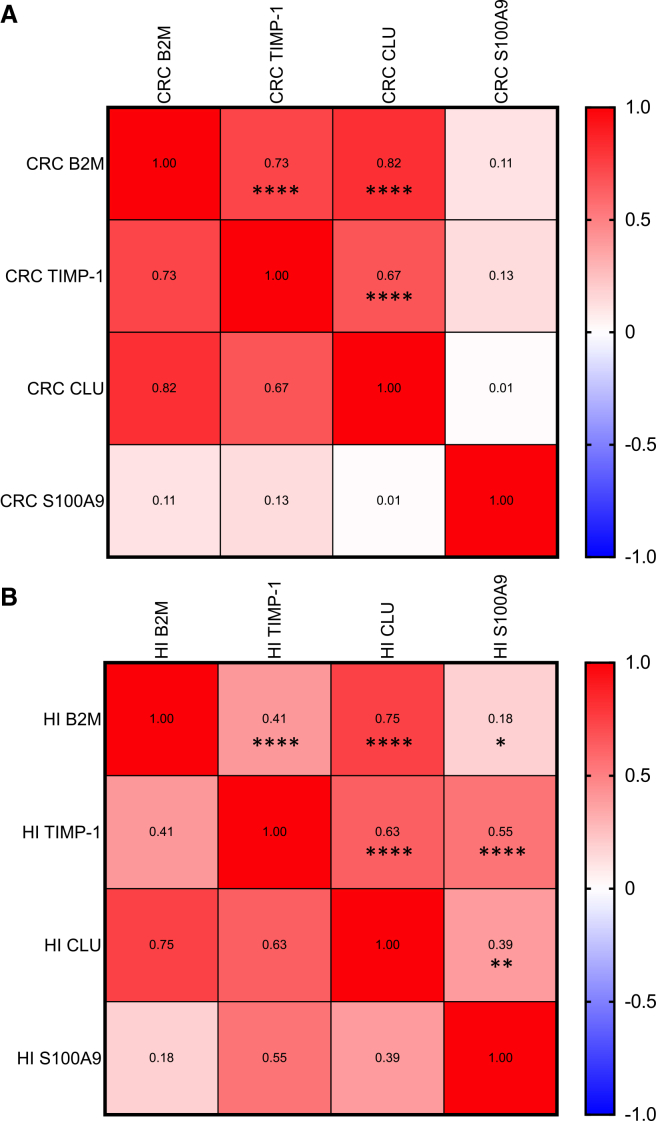
Spearman correlation between each circulating mRNA Spearman correlation between B2M, TIMP-1, CLU, and S100A9 values in CRC (A) and HI (B) patients. The R values and statistical significance of the correlation between each mRNA are shown. ∗*p* < 0.05; ∗∗*p* < 0.01; ∗∗∗*p* < 0.001; ∗∗∗∗*p* < 0.0001.

B2M, TIMP-1, and CLU mRNA levels in HI plasma samples prepared from EDTA tubes (*n* = 171) were compared with those in samples previously prepared from Streck tubes (*n* = 53).^11^ The levels of B2M, TIMP-1, and CLU mRNAs extracted from EDTA tubes were higher than those extracted from Streck tubes (Figures 1A–1C). We therefore hypothesized that some of the 17 mRNAs previously analyzed from Streck plasma samples^11^ but undetectable in HIs might be detectable in EDTA plasma samples. We detected SERPINE1 (Serp1), galectin-3 (GAL3), and S100A9-encoding mRNAs in the plasma of 103 healthy individual (HI) EDTA samples (Figures 2D–2F). In contrast, mRNAs encoding vascular cell adhesion molecule 1, C-reactive protein, angiogenin, collagen type VI alpha 3 chain, transferrin receptor, spondin 2, keratin 19, CEA cell adhesion molecule 5, matrix metallopeptidase 8, matrix metallopeptidase 9, and MET transcriptional regulator MACC1 remained undetectable.

### Study of B2M, TIMP-1, CLU, Serp1, GAL3, and S100A9 circulating mRNA levels in patients with CRC patients, adenomas, and healthy individuals

The clinicopathological characteristics of the patients and HIs are presented in Table 1. We included samples from 178 patients with colorectal adenocarcinoma and 10 patients with adenomas (9 advanced). The metastatic cohort comprised patients with synchronous and metachronous metastases. We analyzed the mRNA levels of B2M, TIMP-1, CLU, Serp1, GAL3, and S100A9 in 178 plasmas of CRC patients in comparison with 171 HI for B2M, TIMP-1, and CLU and 103 HI for Serp1, GAL3, and S100A9 (68 plasmas from HI could not be assayed for Serp1, GAL3, and S100A9 because they were no longer available). As shown in Figure 1 and Table 2, B2M, TIMP-1, CLU, and S100A9 mRNA levels were significantly different between HI and CRC patients (*p* = 0.016, *p* < 0.001, *p* < 0.001 and *p* = 0.0006, respectively), unlike Serp1 and GAL3 mRNA levels. In addition, the levels of the 6 mRNAs were not significantly different between HIs and 10 patients with adenomas.

**Table 1:** Main clinicopathologic characteristics of patients

	CRC (*n* = 178)	HI (*n* = 171)	Adenoma (*n* = 10)
**Gender**			
Male	91 (51.1%)	103 (66.0%)	5 (50.0%)
Female	87 (48.9%)	53 (34.0%)	5 (50.0%)
Not specified		15	
**Age (years)**			
Mean, range	66.6 (33–92)	43.6 (18–69)	57.6 (43–75)
**Primary site**			
Right colon	76 (48.4%)		
Left colon	58 (36.9%)		
Rectum	10 (6.4%)		
Multiple	13(8.3%)		
Not specified	21		
**Stage**			
Advanced adenomas			9
I	36 (20.2%)		
II	46 (25.8%)		
III	44 (24.7%)		
Metastatic (synchronous/metachronous)	52 (48/4) (29.2%)		
**Metastatic sites**			
Liver	33 (63.5%)		
Lung	14 (26.9%)		
Peritoneum	16 (30.8%)		
Distant lymphatic nodes	5 (9.6%)		
Bone	2 (3.8%)		
Other sites	5 (9.6%)

**Table 2:** Median levels and their 95% confidence intervals (CI) were calculated for each group

	HI (*n* = 171)	Stage I (*n* = 36)	Stage II (*n* = 46)	stage III (*n* = 44)	Metastatic (*n* = 52)	Total CRC (*n* = 178)
**B2M**						
Median	7336	7149	9556	11508	11393	10609
SD	16473	23526	10207	20818	19654	19069
p value		0.7798	0.1301	0.0666	0.0057∗∗	0.0157∗
95% CI of median	5800–9046	2965–13026	7368–11809	5822–19529	8563–24934	8553–12116
Confidence level	95%	97%	97%	95%	96%	96%
**TIMP-1**						
Median	291.9	366.5	615	637.5	878.5	632.5
SD	634.5	1520	887.9	975.7	1829	1401
*p* value		0.1628	<0.0001∗∗∗∗	<0.0001∗∗∗∗	<0.0001∗∗∗∗	<0.0001∗∗∗∗
95% CI of median	210–366	196–748	492–716	478–1191	648–1139	554–749
Confidence level	95%	97%	97%	95%	96%	96%
**CLU**						
Median	263	380	1129	892	1141	923
SD	1985	1931	2005	1629	2540	2107
*p* value		0.4264	<0.0001∗∗∗∗	0.0014∗∗	<0.0001∗∗∗∗	<0.0001∗∗∗∗
95% CI of median	95%	97%	97%	95%	96%	96%
confidence level	131–451	114–1013	656–1418	432–1461	443–1941	656–1123
**Serp1**						
Median	5.7	6.5	6	9	6	6
SD	12	14	16	13	12	14
*p* value		0.5265	0.5726	0.2087	0.4446	0.2402
95% CI of median	95%	97%	97%	95%	96%	96%
confidence level	4.7–6.9	3–13	4–8	5–13	3–11	5–8
**GAL3**						
Median	4.3	5	3.5	4.5	3	4
SD	3.9	5.6	4.1	5.4	3	4.6
*p* value		0.3864	0.6678	0.9555	0.0364∗	0.4504
95% CI of median	95%	97%	97%	95%	96%	96%
confidence level	3.3–5	3–7	3–5	3–6	2–4	3–4
**S100A9**						
Median	62	91	94	100	86	94
SD	67	134	349	123	132	208
*p* value		0.0525	0.0178∗	0.0114∗	0.0095∗∗	0.0006∗∗∗
95% CI of median	95%	97%	97%	95%	96%	96%
confidence level	54–75	64–129	70–127	71–125	71–102	81–103
**I-BTC index**						
Median	0.364	0.529	0.654	0.614	0.640	0.620
SD	0.202	0.190	0.168	0.177	0.230	0.199
*p* value		0.0011∗∗	<0.0001∗∗∗∗	<0.0001∗∗∗∗	<0.0001∗∗∗∗	<0.0001∗∗∗∗
95% CI of median	95.4%	97.1%	97.4%	95.1%	96.4%	95.7%
confidence level	0.324–0.422	0.384–0.624	0.603–0.718	0.559–0.677	0.516–0.757	0.579–0.653

Due to the small sample sizes in some subgroups (e.g., stage I, *n* = 36), the actual confidence levels varied slightly as determined by the nonparametric methods. Non-detectable mRNA levels were imputed as half the limit of detection (LOD/2) for statistical analyses. Group comparisons were performed using the Mann-Whitney U test to account for unequal sample sizes and non-normal distributions. ∗*p* < 0.05; ∗∗*p* < 0.01; ∗∗∗*p* < 0.001; ∗∗∗∗*p* < 0.0001.

### Correlations between B2M, TIMP-1, CLU, and S100A9 circulating mRNA levels

**Figure 3:**
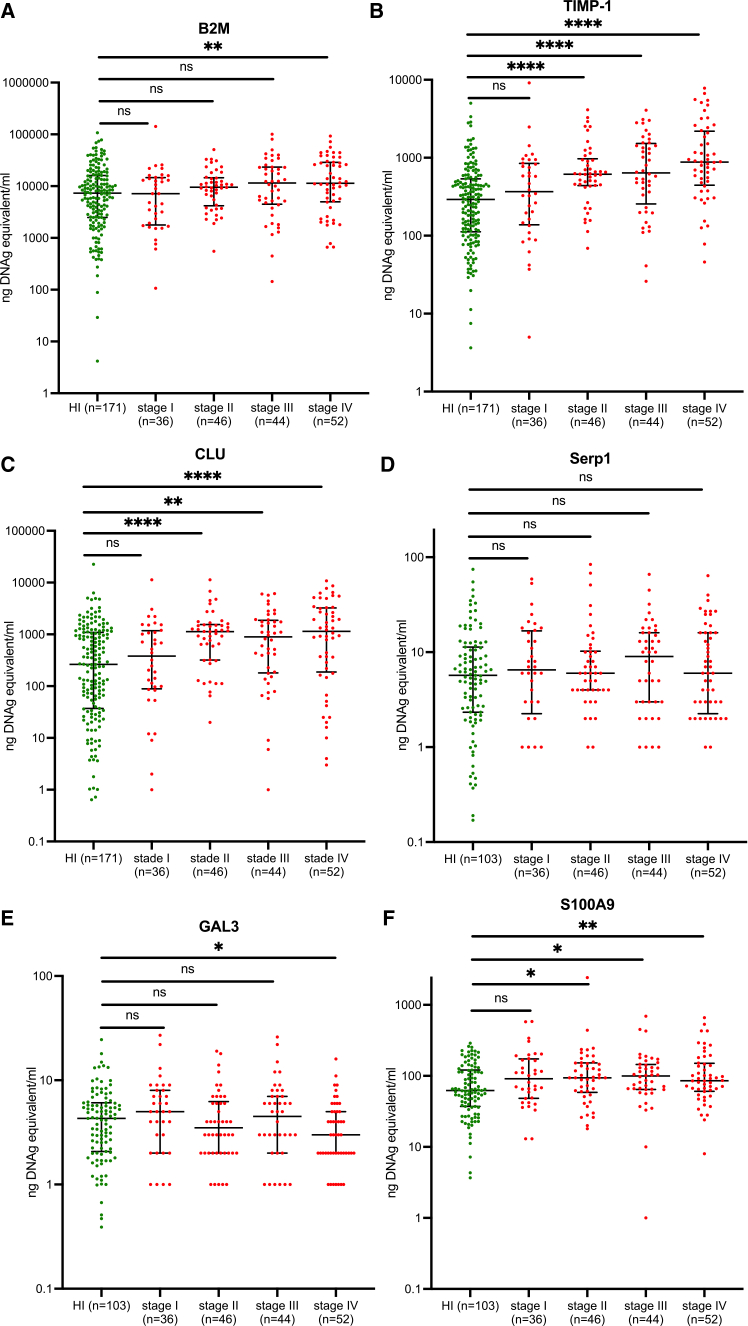
Circulating plasma mRNA at each stage of CRC B2M (A), TIMP-1 (B), CLU (C), Serp1 (D), GAL3 (E), and S100A9 (F) mRNA plasma levels in HI, stage I, stage II, stage III, and IV CRC patients. Medians are shown, and error bars indicate the interquartile range. The results are expressed in ng human genomic DNA (DNAg) equivalent per mL. All values and statistics are given in Table 2. The non-parametric Mann-Whitney test was used, ∗*p* < 0.05; ∗∗*p* < 0.01; ∗∗∗*p* < 0.001; ∗∗∗∗*p* < 0.0001.

Spearman correlations between B2M, TIMP-1, CLU, and S100A9 mRNA values are shown in Figure 2A for CRC (*n* = 178) and Figure 3B for HI (*n* = 103). All mRNAs revealed positive coefficients of correlation (R values). In CRC samples, we observed a weak correlation of S100A9 with other mRNAs (B2M, TIMP-1, and CLU) (Figure 2A). In the HI samples, the correlation of S100A9 was moderate with TIMP-1 and weak with B2M and CLU (Figure 2B). For both HI and CRC, all other correlations were strong or moderate, except for that between B2M and TIMP-1 in HI, which was weak (Figure 2B).

### Associations of B2M, TIMP-1, CLU, Serp1, GAL3, and S100A9 circulating mRNA levels with the stage of CRC

The levels of each mRNA were then analyzed according to the stage of the disease (Figure 3; Table 2). B2M mRNA levels were significantly different (*p* = 0.0057) between metastatic patients (*n* = 52) and HI patients (*n* = 171) (Figure 2A). TIMP-1 (Figure 3B), CLU (Figure 3B) and S100A9 (Figure 3F) mRNA levels were significantly different between HI and stage II (*n* = 46) (*p* < 0.0001, *p* < 0.0001 and 0.0178, respectively), stage III (*n* = 44) (*p* < 0.0001, *p* = 0.0014 and 0.0114, respectively), and metastatic patients (*p* ≤ 0.0001, *p* < 0.0001 and 0.0095, respectively). Serp1 mRNA levels were not significantly different between HIs (*n* = 103) and any of the 4 stages of the disease (Figure 3D). Interestingly, metastatic CRCs (*n* = 52) had a significantly (*p* = 0.0364) lower level of GAL3 than HIs (*n* = 103) (Figure 3E). Regardless of the mRNA analyzed, we did not observe any significant difference between stage I patients (*n* = 36) and HIs.

### Design of a combinatory diagnostic index

**Figure 4:**
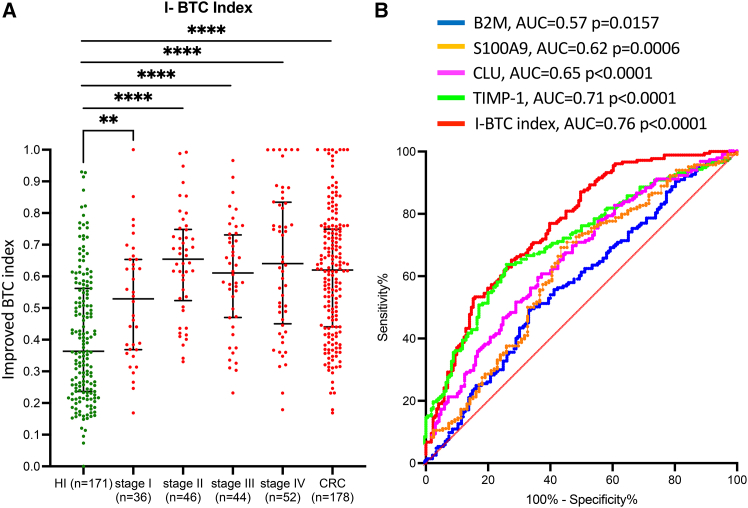
Diagnostic performance of the I-BTC index (A) D-index values for HI, stage I, stage II, stage III, and stage IV CRC patients. Medians are shown, and error bars indicate the interquartile range. The non-parametric Mann-Whitney test was used, ∗*p* < 0.05; ∗∗*p* < 0.01; ∗∗∗*p* < 0.001; ∗∗∗∗*p* < 0.0001. (B) Receiver operating characteristic curve showing the diagnostic power of the B2M, TIMP-1, CLU, S100A9, and I-BTC index to discriminate between plasma samples from patients with CRC and HIs. All values and statistics are provided in Table 2.

Previously, we defined the BTC index, which represents the sum of the B2M, TIMP-1, and CLU values for diagnosing metastatic CRC. In this study, we used a similar approach but excluded the Serp1 and GAL3 mRNAs, as they have low diagnostic value when assessed individually (Figures 3D and 3E). We performed logistic regressions to combine the individual values of B2M, TIMP-1, CLU, and S100A9. The optimal combination, named the improved BTC index (I-BTC index), included B2M, TIMP-1, and CLU. The I-BTC index was established via the formula mentioned in the materials and methods section. All the specifications of the logistic regression are described in Table S1, including that 67.25% of HI patients and 68.54% of CRC patients were correctly classified. The median I-BTC index for HI patients was 0.364, whereas that for CRC patients was 0.62 (Table 2). As shown in Figure 4A and Table 2, the discriminatory power of the I-BTC index is significantly greater than that of the individual B2M, TIMP-1, and CLU values (Figures 1 and 2), demonstrating a clear and significant distinction between HI and CRC, regardless of disease stage. In addition, the receiver operating characteristic (ROC) curve for the I-BTC index outperformed those of B2M, TIMP-1, CLU, or S100A9, with an area under the curve (AUC) of 0.762 (Figure 4B). I-BTC index clinical diagnostic parameters including the area under the ROC curve (AUC), the optimal cutoff value calculated using Youden’s index, sensitivity, specificity, and positive and negative predictive values (PPV/NPV), are listed in Table 3. The I-BTC index demonstrated varying diagnostic performance across colorectal cancer stages, with AUC values ranging from 0.67 in stage I to 0.81 in stage II. For the entire cohort (CRC, *n* = 178), the overall AUC was 0.76. The 95% confidence intervals for these AUCs were consistently narrow—such as 0.75 to 0.87 for stage II—indicating precise estimates. Sensitivity peaked at 87.8% for combined early stages (stages I + II), highlighting the index’s potential for early detection. Specificity, on the other hand, was highest in stage IV (84.8%). The PPV varied widely, from 24.4% in stage I to 71.2% across all CRC cases, while the NPV remained robust (>84% for all stages). Finally, the Youden index was maximized in stage IV (0.6153), suggesting an optimal balance between sensitivity and specificity at this advanced stage.

**Table 3:** key clinical diagnostic parameters of the I-BTC index

Stage	AUC	Std. Error	95% CI	youden	Sens. %	95% CI	Spe. %	95% CI	PPV %	NPV %
I (*n* = 36)	0.67	0.04	0.58–0.76	0.3463	83.33	68.11–92.13	45.61	38.33–53.09	24.4	92.9
II (*n* = 46)	0.81	0.03	0.75–0.87	0.5875	69.57	55.19–80.92	80.12	73.50–85.41	48.5	90.7
III (*n* = 44)	0.76	0.04	0.69–0.84	0.5339	70.45	55.78–81.84	72.51	65.38–78.66	39.7	84
IV (*n* = 178)	0.78	0.04	0.71–0.85	0.6153	55.77	42.34–68.40	84.8	78.65–89.41	52.7	86.3
I + II (*n* = 82)	0.75	0.03	0.69–0.81	0.3658	87.8	78.99–93.24	50.29	42.87–57.70	45.9	90.5
CRC (*n* = 178)	0.76	0.03	0.71–0.81	0.5336	65.17	57.92–71.78	72.51	65.38–78.66	71.2	66.7

### Pre- and postsurgery levels of B2M, TIMP-1, CLU, and S100A9 circulating mRNAs in stage II and III CRC patients

**Figure 5:**
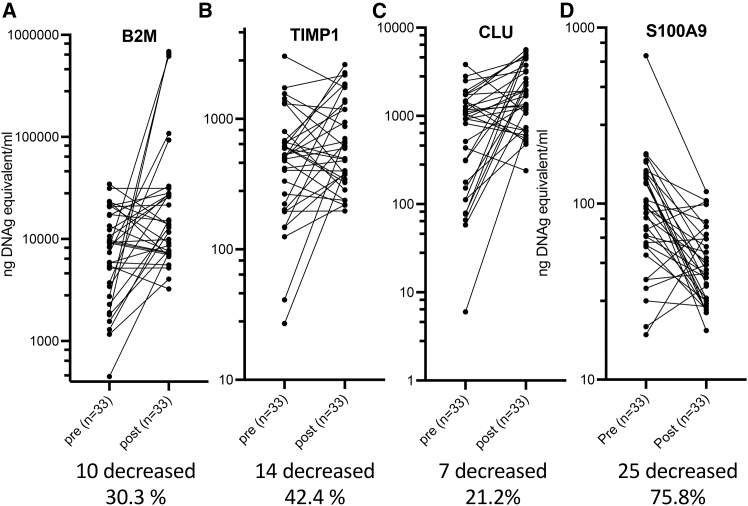
Pre- and postsurgery circulating plasma mRNA Pre- and postsurgery B2M (A), TIMP-1 (B), CLU (C), and S100A9 (D) mRNA levels in stage II and III patients. The results are expressed in ng human genomic DNA (DNAg) equivalent per mL. See also Table S2.

B2M, TIMP-1, CLU, and S100A9 mRNAs were assessed in plasma collected from 33 patients classified as stage II or III, before and after surgery (Figure 5). Patients seen in consultation for postoperative sampling showed no obvious signs of infection or complications. The average interval between the two samples was 24 days, with a median of 22 days. Levels of B2M, TIMP-1, and CLU increased in the majority of patients (Figures 5A–5C), whereas S100A9 levels decreased in the large majority (*n* = 25) (Figure 5D). The median B2M and CLU values were significantly higher in post-surgery samples (*p* = 0.008 and *p* = 0.0006, respectively), whereas the median S100A9 value was lower in post-surgery samples (*p* < 0.0001) (Table S2). Surprisingly, the median S100A9 level after surgery was significantly lower than the median of HI (*p* = 0.0103).

## Discussion

Colonoscopy with biopsy remains the gold standard for diagnosing colorectal precancerous and cancerous tumors,^14^ but given its drawbacks (colon cleansing, invasiveness, cost, etc.), there is an urgent need to develop new and less invasive approaches that may increase patient adherence and enable better selection of those requiring this invasive examination. Some studies have already demonstrated that blood-based tests (using ctDNA as the main analyte) improve colorectal screening in patients who declined colonoscopy and FITs as the first option,^15^ but compared with other approved screening strategies, they remain the least cost-effective strategy in terms of current price and performance.^10^^,^^16^^,^^17^^,^^18^ Therefore, large efforts have to be made not only to reduce their costs but also to improve their sensitivity, especially for detecting advanced adenomas, if they want to substitute the established methods.^19^

The development of our RNA-2C method aims to address some of these challenges. By quantifying some circulating mRNAs, we previously demonstrated their ability to distinguish between patients with metastatic CRC and HIs via blood samples collected in Streck cell-free DNA BCT tubes. To further study its applicability in the diagnostic setting, we analyzed patients with different stages of the disease and used archived plasma extracted from blood samples collected in EDTA tubes, a much more widely used material. We show that EDTA tubes are suitable for the RNA-2C method and that the quantity of purified and analyzed mRNAs is greater than that of plasma mRNAs extracted from blood samples collected in Streck tubes. This observation has not been reported for circulating DNA,^12^ and we currently have no clear explanation for this discrepancy. As previously observed with Streck mRNA samples, the levels of TIMP-1 and CLU mRNA in EDTA samples were significantly greater in metastatic patients than in HI patients according to the Mann-Whitney test. In contrast, for B2M mRNAs, the difference between stages IV and HI was less significant in EDTA samples than in Streck samples.^11^ Thus, preanalytical conditions are critical and must be rigorously applied to enable quantitative comparisons of studies. Another explanation could be that the storage of patient plasma at −80°C (up to 8 years for some patients) may alter the stability of some of the mRNAs, whereas HI plasma is stored for much shorter periods. This question is inherent to retrospective studies and will be resolved as soon as we will be able to analyze freshly harvested patients. However, since CRC patients have higher mRNA levels than HIs do globally, we believe that this phenomenon is likely not critical and that shortening the storage period will probably improve the results. We also found that plasma mRNAs remain stable in plasma samples prepared up to 3 h after blood collection, which is consistent with published data showing that plasma mRNAs are protected within circulating extracellular vesicles.^13^ In line with these studies, we found that purified mRNA added to plasma could not be analyzed using the RNA-2C method due to its degradation by plasma RNases (not shown).

Owing to the higher levels of mRNAs in EDTA plasma samples than in Streck samples, we were able to detect 3 additional mRNAs, i.e., Serp1, GAL3, and S100A9, whose levels are accordingly lower than those of B2M, TIMP-1, and CLU. Physiologically, it is now clear that plasma contains some mRNAs whose exact origin is unknown.^20^^,^^21^^,^^22^ It is rational to assume that the majority of mRNAs are derived from blood cells, but it is quite possible that a given mRNA can originate from several organs, including the blood itself.

A comparison of the plasma from 178 CRC patients with HI revealed significant differences in B2M, TIMP-1, CLU, and S100A9 mRNA levels. When analyzing the levels of these mRNAs across disease stages, we observed a significant increase in the expression of several transcripts as early as stage II, particularly for TIMP-1, CLU, and S100A9. This early-stage elevation suggests their potential utility as biomarkers for detecting colorectal cancer progression at relatively initial phases. In contrast, B2M levels only rose significantly in stage IV, indicating a more limited role in early detection. However, the wide confidence intervals observed for certain biomarkers, notably CLU and TIMP-1, highlight substantial intra-group variability. This variability may reflect biological heterogeneity or the relatively small sample sizes in some subgroups (e.g., stage I, *n* = 36). Validation in a larger, independent cohort would be essential to refine these estimates and confirm the robustness of these findings in a clinical setting. These differences suggest that the presence of a colorectal tumor leads to an increase in the levels of certain mRNAs in the plasma without knowing whether they truly originate from the tumor cells themselves. This increase in circulating mRNAs could also results from tumor-induced inflammation. However, no significant increase was observed in the plasma of patients with advanced adenomas, which should be interpreted with caution given the small sample size (*n* = 10). Nevertheless, this weakens the hypothesis of tumor-induced inflammation without completely ruling it out.

With respect to diagnostic performance, no single circulating mRNA provided a satisfactory predictive capacity except for TIMP-1, which reached an AUC of 0.71. This is mainly explained by the low sensitivity of each biomarker for detecting the early stages of the disease. We must acknowledge that even though we have sufficient data demonstrating that dysregulation of the genes encoding these RNAs is related to colorectal carcinogenesis, we cannot say that a change in expression occurs in the early stages of the process. For example, several studies have shown that TIMP-1 mRNA is overexpressed in CRC tissue compared with normal tissue and that the differential expression is more pronounced in tumors with positive lymph nodes and distant organ spread. In addition, TIMP-1 has been proposed as a prognostic biomarker in gastrointestinal cancer,^21^ suggesting its role in the acquisition of characteristics associated with more advanced diseases, such as invasion and migration. To improve the performance of our test, we performed several combinations of available biomarkers via logistic regression and found that combining the values of B2M, TIMP-1, and CLU-encoding RNAs yielded the best result. This formula was further used to calculate a diagnostic index (the I-BTC index), which yields a highly significant difference between all stages and the HI. The I-BTC index also provides information on the probability of suffering from CRC, with values varying between 0 and 1 so that a value of 1 indicates a 100% risk of suffering from the disease, whereas 0 indicates no risk. Logistic regressions that included S100A9 alongside other circulating mRNAs did not improve the results, which could be due to the weak correlation between S100A9 and the other mRNAs. The I-BTC index demonstrates strong discriminatory potential, particularly in early-stage colorectal cancer, with an AUC of 0.81 in stage II—a critical advantage for early diagnosis. Its high sensitivity (87.8%) in combined stages I + II suggests a valuable tool for minimizing false negatives, while specificity reaches 84.8% in stage IV, offering reliable confirmation of advanced disease. However, the moderate PPV—especially in stage I (24.4%)—shows that biomarker-based diagnosis still struggles to accurately identify true positives when disease prevalence is low. In contrast, the consistently high negative predictive value (NPV >84%) across all stages provides robust reassurance for ruling out disease, particularly in early-stage screening. The wider confidence intervals observed in smaller subgroups (e.g., stage I, *n* = 36) emphasize the need for validation in larger, independent cohorts. Future work should also focus on optimizing diagnostic thresholds—leveraging the Youden index—to enhance PPV in early stages, where clinical impact is greatest. Notably, the I-BTC index outperforms traditional biomarkers such as CEA and CA19-9,^23^ which have shown lower AUCs in comparable settings. This positions I-BTC as a promising alternative for improving colorectal cancer stratification and early detection even though the global diagnostic performance of our index remains lower than that of other blood-based tests currently used in CRC screening strategies. The most recent assays, which are based on panels of cirDNA features (e.g., Shield test ™) or cirDNA features plus plasma proteins (e.g., Freenome test), achieve sensitivities for colorectal cancer between 80% and 83%, with specificities of more than 90% in average risk populations.^20^^,^^23^ Some alternative circulating mRNA-based methods have also been proposed for diagnostic purposes,^24^^,^^25^^,^^26^ some of which identify transcriptome profiles with better AUCs than ours does, although they require prospective validation. In addition, they are more costly and less easy to implement. In any case, the use of biomarkers on the basis of the expression pattern of extracellular RNAs seems to be a promising avenue to optimize diagnostic accuracy, particularly for early stages, as evidenced by the Colosense test. In the CRC-PREVENT phase 3 clinical study, this multitarget stool test, which incorporates a commercial FIT and the concentrations of 8 RNA transcripts, showed 46% sensitivity for detecting advanced adenomas in 8920 individuals 45 years and older.^27^ This value was significantly higher than the results of FIT alone (46% vs. 29%; *p* < 0.001) and, to date, the best performance ever reached by the emerging assays tested prospectively in this setting.^28^

Unlike S100A9, we found that most other circulating biomarkers increased in post-resection samples, while the presumed main source was removed. This raises the question of their tumor specificity and their origin before being shed into the systemic circulation and could reinforce our other hypothesis that the selected circulating RNAs may originate from the microenvironment—rather than from tumor cells—particularly from immune cells. As an example, Jin et al. recently demonstrated, through transcriptomic and single-cell data analysis, that TIMP1 is highly expressed by neutrophils in CRC tissue compared with normal tissue.^29^ Moreover, they identified TIMP1 as a pivotal gene involved in neutrophil extracellular trap (NET) formation. In parallel, our team, studying the dynamics of total circulating DNA (cir-nDNA) over an extended post-surgery period in patients who underwent curative resection for stage III colon cancer, reported that the median concentration of cir-nDNA remains greater than the pre-surgery value and is highly associated with NET markers but is not correlated with relapse risk or the use of adjuvant chemotherapy.^30^ Thus, the increase in TIMP1 gene expression observed in our study could simply reflect NET production triggered by the presence of the tumor but also by surgery. An analysis of patients who would undergo bowel surgery for reasons other than tumor resection would be interesting to determine whether surgery truly has an impact on circulating mRNA levels.

This study has several other limitations, particularly the small sample size of each group and its retrospective nature probably impacted the technical and clinical validity of our test. Independent prospective studies are essential before it can be implemented in clinical practice.

However, this study lays the groundwork for the future development of a noninvasive and easily accessible screening method based on circulating mRNAs. To date, we have focused on 17 empirically selected mRNAs, but we plan to identify additional circulating mRNAs to improve our test, using a more systematic method, especially in patients with precancerous and early-stage cancerous lesions. An additional strategy would be to combine them with other liquid biopsy parameters, such as circulating DNA. For example, a blood-based test combining DNA methylation, 5′ end motif, copy number variation and genetic mutations by logistic regression has recently been proposed for the detection of CRC,^31^ and mRNA analysis could be an additional parameter of such a comprehensive test.

The present study confirms that the RNA-2C method is a practical tool for detecting and quantifying circulating mRNAs. It could be useful for the early screening for CRC as the combination of B2M, TIMP-1, and CLU mRNA values make it possible to distinguish CRC patients from HIs.

### Limitations of the study

Our study highlights the diagnostic potential of I-BTC for CRC but has some limitations. First, the age gap between healthy controls (mean age 43) and CRC patients (66) could confound the results, as I-BTC may be linked to immune and inflammatory processes. Although we stratified the healthy cohort, we did not perform age-adjusted analyses or matching. Second, the lack of internal validation (e.g., cross-validation) limits the generalizability of our performance metrics (AUC, sensitivity, and specificity), which may be overestimated due to model complexity. Finally, the small adenoma cohort (*n* = 10) and the absence of clinically relevant controls, such as benign inflammatory conditions or advanced adenomas, restrict our ability to assess I-BTC’s specificity for early detection. Addressing these limitations through age-matched cohorts, robust validation, and diverse control groups will be key to confirming I-BTC’s clinical utility in larger prospective studies.

## Resource availability

### Lead contact

Requests for additional information and resources should be directed to the contact manager, who will provide the necessary replies, Philippe Blache (philippe.blache@inserm.fr).

### Materials availability

This study did not result in the development of any new specific reagents.

### Data and code availability

• All data reported in this paper will be shared by the lead contact upon request.
• This paper does not report original code.
• Any additional information required to reanalyze the data reported in this paper is available from the lead contact upon request.

## Acknowledgments

We thank Etablissement Français du Sang, Montpellier, France, for providing blood samples from healthy donors. We are very grateful to the Biological Resource Center of the Montpellier Cancer Institute (CRB-ICM) for providing us with the patient samples. This work was supported by SIRIC Montpellier Cancer, grant reference: INCa_Inserm_DGOS_12553, 10.13039/501100004099Ligue Contre le Cancer Comité Gard, ANR RHU REVEAL A.THIERRY (G22002FF / RGE22001FFA) and KIM Biomarkers & Therapy Université de Montpellier.

## Author contributions

Conceptualization, data curation, formal analysis, project administration, resources, writing – review and editing, T.M.; data curation, formal analysis, investigation, M.G.; data curation, formal analysis, investigation, L.V.; data curation, formal analysis, resources, S.T.; data curation, formal analysis, resources, B.P.; data curation, formal analysis, resources, E.P.; data curation, formal analysis, resources, G.L.; data curation, formal analysis, resources, E.L.-C.; data curation, formal analysis, resources, M.Y.; data curation, formal analysis, resources, A.R.T.; data curation, formal analysis, resources, writing – review and editing, C.P.; conceptualization, data curation, formal analysis, funding acquisition, project administration, resources, writing – original draft, writing – review and editing, P.B.

## Declaration of interests

The authors declare no competing interest.

## Declaration of generative AI and AI-assisted technologies in the writing process

During the preparation of this work the author(s) used MISTRAL (https://chat.mistral.ai/chat) in order to improve the syntax of the manuscript. After using this tool/service, the author(s) reviewed and edited the content as needed and take(s) full responsibility for the content of the publication.

## STAR★Methods

### Key resources table

**Table:** 

REAGENT or RESOURCE	SOURCE	IDENTIFIER
**Biological samples**		

Healthy individuals blood samples	This paper	N/A
Colorectal cancer patients blood samples	This paper	N/A

**Oligonucleotides**		

All oligonucleotides used in this paper have been previously described	M. Grosgeorges et al., Sci Rep 13 (2023) 2739.	N/A

**Software and algorithms**		

GraphPad Prism10 version 10.6.1	Graphpad	https://www.graphpad.com/

**Other**		

Plasma/Serum RNA/DNA Purification Mini Kit	Norgen	reference 55,200
QuantiTect Reverse Transcription Kit	Qiagen	reference 20,531
iQTM SYBR® Green Supermix	Bio-Rad	reference 1708882
human genomic DNA	Promega	reference G1471

### Experimental model and study participant details

#### Patients and cohorts

Plasmas (stored at −80°C) were supplied by the Biological Resource Center of the Montpellier Cancer Institute (CRB-ICM). They were obtained from patients with colorectal tumors diagnosed at different stages of the disease prior to any treatment and prospectively included in 3 clinico-biological databases: BCB COLON-LR and Institutional BCB COLON (NCT03976960;ID-RCB: 2014-A00033-44), which recruit patients with stage I to IV CRC and encompass blood sample collection before and after primary tumor surgery; BCB Cbio-dig (NCT03978078;ID-RCB:2016-A00083-48), which recruits patients with unresectable metastatic CRC prior to the initiation of 1st-line systemic anticancer therapies and encompasses blood sample collection before the start of treatment. Patients were informed about the use of biological resources, and all provided their consent. HI blood samples were provided by the “Etablissement Français du Sang” Center in Montpellier, France (Agreement EFS-PM n. 21PLER2015-0013).

The clinicopathological features of the patients and HIs are summarized in Table 1. Our cohort comprised samples from 178 individuals with colorectal adenocarcinoma and 10 with adenomas (9 advanced). Metastatic cases included both synchronous and metachronous metastases.

Age-related changes in gene expression and systemic inflammation are well documented, which led us to wonder whether age might influence circulating mRNA levels. We focused on TIMP-1 mRNAs levels in HIs aged under or over 50 years of age, given that the risk of developing CRC increases significantly after the age of 50, which is also the recommended age in France to start the screening program in the middle risk population. The two groups had similar median levels for TIMP-1 mRNA (243.9 for the youngest individuals (*n* = 90) vs. 264.9 for the oldest individuals (*n* = 50) (Figure S1). We then analyzed circulating mRNAs (B2M, TIMP1, CLU, and S100A9) in HI stratified by age groups (≤40, 40–60, and ≥60 years) and found no statistically significant differences between these age groups either (Figure S2). We also observed that the level of TIMP-1 in the plasma of HIs was not influenced by gender (women: median = 259.5; *n* = 53; men: median = 250.4; *n* = 103; *p* = 0.566) (Figure S3) as was also the case for the other mRNAs studied (data not shown).

#### Ethical consideration

The Agence Nationale de Sécurité du Médicament et des Produits de Santé and the Comités de Protection des Personnes approved this study according to the French national regulatory requirements. Blood samples from HIs were provided by the “Etablissement Français du Sang, the blood transfusion center of Montpellier, France” (Agreement EFS-PM n. 21PLER2015-0013). All procedures were performed in compliance with the applicable guidelines and regulations.

### Method details

#### Blood sample collection

Blood was collected in ethylenediaminetetraacetic acid (EDTA) tubes (the standard in clinical practice), and plasma was extracted within 3 h by centrifugation (1200 × g) for 10 min at 4°C and stored at −80°C until analysis. We previously showed that mRNAs are stable for several days in blood collected in Streck tubes.^11^ To determine their stability in blood collected in EDTA tubes, plasmas from 2 individuals sampled in those tubes were prepared immediately (within 15 min), 1 h later and 3 h later and analyzed for B2M, TIMP-1 and CLU mRNAs. We observed no significant variation in mRNA levels, regardless of the time elapsed between blood collection and plasma preparation (Figure S4). Given that, the time between blood collection and plasma preparation did not exceed 3 h for all analyses carried out in this study, we assume that this time interval did not affect mRNA quantification. Furthermore, only plasma samples showing no hemolysis or significant turbidity were analyzed.

#### Circulating RNA extraction, reverse transcription and quantification of mRNA

The RNA-2C method, along with its detection limit, linearity, and the nucleotide primers used, has already been described.^11^ Briefly, RNA was purified from plasma (200 μL) via the Plasma/Serum RNA/DNA Purification Mini Kit (Norgen, reference 55,200). Reverse transcription was performed with the QuantiTect Reverse Transcription Kit (Qiagen; reference 20,531). QPCR was performed with iQTM SYBR Green Supermix (Bio-Rad) and a LightCycler 480 apparatus (Roche). The absolute quantification was performed with human genomic DNA (DNAg) (Promega, reference G1471), and the results are expressed in ng DNAg equivalent. To ensure that the prepared cDNA was free from DNA contamination, each run included a control condition in which primers designed to specifically bind to DNAg sequences and not to cDNA were used.^11^

### Quantification and statistical analysis

To compare the patients circulating mRNA concentrations at different stage of the disease or at different time points, we utilized the non-parametric Mann–Whitney test. Medians are shown on figures, and error bars indicate the interquartile range. ∗*p* < 0.05 (significant); ∗∗*p* < 0.01 (very significant); ∗∗∗*p* < 0.001 (extremely significant); ∗∗∗∗*p* < 0.0001 (extremely significant).

To evaluate the correlation between the biomarker plasma levels, Spearman correlation coefficients (CCs) were calculated. The correlation is negligible if it is less than 0.3, weak if the CC is between 0.3 and 0.5, moderate if the CC is between 0.5 and 0.8 and strong if the CC is > 0.8.^32^

Using Graph Pad Prism software, we designed a 3-mRNA model (B2M, TIMP-1 and CLU) diagnostic panel through logistic regression via the following formula: I-BTC index = 1/(1 + exp(-(-0.8944-0,00009168∗B2M+0.00852∗TIMP1-0.003053∗CLU+0.000000001875∗ B2M ˆ2–0.000003282∗ TIMP1ˆ2 + 0.000000337∗ CLU ˆ2–0.00000000000001125∗ B2M ˆ3 + 0.0000000004891∗ TIMP1ˆ3–0.00000000001278∗ CLU ˆ3–0.007588∗sqrt(B2M)- 0.1187∗ sqrt (TIMP1)+ 0.1497∗ sqrt (CLU))). The properties of this logistic regression are detailed in Table S1.

In order to assess the predictive value of circulating mRNA concentrations, receiver operating characteristic (ROC) curves were plotted and the area under the curve (AUC) associated with circulating mRNA levels, along with its 95% confidence interval were assessed. The optimal cut-off value was determined using the Youden index, calculated as the sum of sensitivity and specificity, minus one. The positive predictive value (PPV) of the I-BTC index reflects the probability that individuals with a positive test result actually have the disease. It was calculated as the ratio of true positives to the total number of positive results (true positives + false positives). The negative predictive value (NPV) indicates the probability that individuals with a negative test result are actually free of disease; it is calculated as the ratio of true negatives to the total number of negative results (true negatives + false negatives).

## References

[1] Siegel R.L., Giaquinto A.N., Jemal A. (2024). Cancer statistics, 2024. CA Cancer J. Clin..

[2] Nors J., Gotschalck K.A., Erichsen R., Andersen C.L. (2024). Incidence of late recurrence and second primary cancers 5–10 years after non-metastatic colorectal cancer. Int. J. Cancer.

[3] Levin B., Lieberman D.A., McFarland B., Smith R.A., Brooks D., Andrews K.S., Dash C., Giardiello F.M., Glick S., Levin T.R. (2008). Screening and surveillance for the early detection of colorectal cancer and adenomatous polyps, 2008: a joint guideline from the American Cancer Society, the US Multi-Society Task Force on Colorectal Cancer, and the American College of Radiology. CA Cancer J. Clin..

[4] Ibáñez-Sanz G., Milà N., Vidal C., Rocamora J., Moreno V., Sanz-Pamplona R., Garcia M., on behalf of the MSIC-SC research group (2021). Positive impact of a faecal-based screening programme on colorectal cancer mortality risk. PLoS One.

[5] Novotny S.A., Rodrigo Amador V.A., Seguí Orejuela J., López-Pineda A., Quesada J.A., Pereira-Expósito A., Carratalá-Munuera C., Hernandis Villalba J., Gil-Guillén V.F. (2024). Prognostic Study of Colorectal Cancer: Differences between Screen-Detected and Symptom-Diagnosed Patients. Cancers.

[6] Sedani A.E., Gomez S.L., Lawrence W.R., Moore J.X., Brandt H.M., Rogers C.R. (2025). Social Risks and Nonadherence to Recommended Cancer Screening Among US Adults. JAMA Netw. Open.

[7] Scherbakov D., Heider P.M., Wehbe R., Alekseyenko A.V., Lenert L.A., Obeid J.S. (2025). Using large language models for extracting stressful life events to assess their impact on preventive colon cancer screening adherence. BMC Public Health.

[8] Zhou M., Wu Y., Wang D., Cheng F. (2024). Information needs for cancer screening and associated factors of information-seeking behaviour: a qualitative systematic review. BMC Public Health.

[9] Doubeni C.A., Fedewa S.A., Levin T.R., Jensen C.D., Saia C., Zebrowski A.M., Quinn V.P., Rendle K.A., Zauber A.G., Becerra-Culqui T.A. (2019). Modifiable Failures in the Colorectal Cancer Screening Process and Their Association With Risk of Death. Gastroenterology.

[10] Ferrari A., Neefs I., Hoeck S., Peeters M., Van Hal G. (2021). Towards Novel Non-Invasive Colorectal Cancer Screening Methods: A Comprehensive Review. Cancers (Basel).

[11] Grosgeorges M., Picque Lasorsa L., Pastor B., Prévostel C., Crapez E., Sanchez C., Frayssinoux F., Jarlier M., Pezzella V., Monard L. (2023). A straightforward method to quantify circulating mRNAs as biomarkers of colorectal cancer. Sci. Rep..

[12] Diaz I.M., Nocon A., Held S.A.E., Kobilay M., Skowasch D., Bronkhorst A.J., Ungerer V., Fredebohm J., Diehl F., Holdenrieder S., Holtrup F. (2023). Pre-Analytical Evaluation of Streck Cell-Free DNA Blood Collection Tubes for Liquid Profiling in Oncology. Diagnostics.

[13] Kim H.J., Rames M.J., Goncalves F., Kirschbaum C.W., Roskams-Hieter B., Spiliotopoulos E., Briand J., Doe A., Estabrook J., Wagner J.T. (2023). Selective enrichment of plasma cell-free messenger RNA in cancer-associated extracellular vesicles. Commun. Biol..

[14] Rex D.K. (2025). Colonoscopy Remains an Important Option for Primary Screening for Colorectal Cancer. Dig. Dis. Sci..

[15] Liang P.S., Zaman A., Kaminsky A., Cui Y., Castillo G., Tenner C.T., Sherman S.E., Dominitz J.A. (2023). Blood Test Increases Colorectal Cancer Screening in Persons Who Declined Colonoscopy and Fecal Immunochemical Test: A Randomized Controlled Trial. Clin. Gastroenterol. Hepatol..

[16] Chuang M.P.-C., Chiu H.-M. (2025). Does Colonoscopy as a First Screening Test Still Make Sense?—Counterpoint. Dig. Dis. Sci..

[17] Aziz Z., Wagner S., Agyekum A., Pumpalova Y.S., Prest M., Lim F., Rustgi S., Kastrinos F., Grady W.M., Hur C. (2023). Cost-Effectiveness of Liquid Biopsy for Colorectal Cancer Screening in Patients Who Are Unscreened. JAMA Netw. Open.

[18] Nascimento De Lima P., Matrajt L., Coronado G., Escaron A.L., Rutter C.M. (2025). Cost-Effectiveness of Noninvasive Colorectal Cancer Screening in Community Clinics. JAMA Netw. Open.

[19] Ladabaum U., Mannalithara A., Weng Y., Schoen R.E., Dominitz J.A., Desai M., Lieberman D. (2024). Comparative Effectiveness and Cost-Effectiveness of Colorectal Cancer Screening With Blood-Based Biomarkers (Liquid Biopsy) vs Fecal Tests or Colonoscopy. Gastroenterology.

[20] Shaukat A., Burke C.A., Chan A.T., Grady W.M., Gupta S., Katona B.W., Ladabaum U., Liang P.S., Liu J.J., Putcha G. (2025). Clinical Validation of a Circulating Tumor DNA-Based Blood Test to Screen for Colorectal Cancer. JAMA.

[21] Qin L., Wang Y., Yang N., Zhang Y., Zhao T., Wu Y., Jiang J. (2021). Tissue inhibitor of metalloproteinase-1 (TIMP-1) as a prognostic biomarker in gastrointestinal cancer: a meta-analysis. PeerJ.

[22] Zeng Z.S., Cohen A.M., Zhang Z.F., Stetler-Stevenson W., Guillem J.G. (1995). Elevated tissue inhibitor of metalloproteinase 1 RNA in colorectal cancer stroma correlates with lymph node and distant metastases. Clin. Cancer Res..

[23] Chung D.C., Gray D.M., Singh H., Issaka R.B., Raymond V.M., Eagle C., Hu S., Chudova D.I., Talasaz A., Greenson J.K. (2024). A Cell-free DNA Blood-Based Test for Colorectal Cancer Screening. N. Engl. J. Med..

[24] Li M., Yu Y., Liu G., Zhang S., Luo C., Hu S., Wan S., Zhao L. (2025). A promising frontier of circulating messenger RNA in liquid biopsy: From mechanisms to clinical applications. Int. J. Cancer.

[25] Tsang H.F., Pei X.M., Wong Y.K.E., Wong S.C.C. (2024). Plasma Circulating mRNA Profile for the Non-Invasive Diagnosis of Colorectal Cancer Using NanoString Technologies. Int. J. Mol. Sci..

[26] Xue V.W., Cheung M.T., Chan P.T., Luk L.L.Y., Lee V.H., Au T.C., Yu A.C., Cho W.C.S., Tsang H.F.A., Chan A.K., Wong S.C.C. (2019). Non-invasive Potential Circulating mRNA Markers for Colorectal Adenoma Using Targeted Sequencing. Sci. Rep..

[27] Barnell E.K., Wurtzler E.M., La Rocca J., Fitzgerald T., Petrone J., Hao Y., Kang Y., Holmes F.L., Lieberman D.A. (2023). Multitarget Stool RNA Test for Colorectal Cancer Screening. JAMA.

[28] Mannucci A., Goel A. (2024). Stool and blood biomarkers for colorectal cancer management: an update on screening and disease monitoring. Mol. Cancer.

[29] Jin Y., Liao L., Chen Q., Tang B., Jiang J., Zhu J., Bai M. (2025). Multi-omics analysis reveals that neutrophil extracellular traps related gene TIMP1 promotes CRC progression and influences ferroptosis. Cancer Cell Int..

[30] Mirandola A., Kudriavtsev A., Cofre Muñoz C.I., Navarro R.C., Macagno M., Daoud S., Sanchez C., Pastor B., Pisareva E., Marin M.S. (2024). Post-surgery sequelae unrelated to disease progression and chemotherapy revealed in follow-up of patients with stage III colon cancer. EBioMedicine.

[31] Gao Y., Cao D., Li M., Zhao F., Wang P., Mei S., Song Q., Wang P., Nie Y., Zhao W. (2024). Integration of multiomics features for blood-based early detection of colorectal cancer. Mol. Cancer.

[32] Schober P., Boer C., Schwarte L.A. (2018). Correlation Coefficients: Appropriate Use and Interpretation. Anesth. Analg..

