# Supplementary material for: An exploratory study of circulating cell-free mRNAs across colorectal cancer stages
Source: iScience. 2026 Jun 17;29(7):116398. doi: 10.1016/j.isci.2026.116398 (PMC13310942; doi:10.1016/j.isci.2026.116398)
Supplement: Document S1. Figures S1–S4 and Tables S1 and S2 [file mmc1.pdf]

iScience, Volume *29*

Supplemental information

An exploratory study of circulating cell-free mRNAs across colorectal cancer stages

Thibault Mazard, Marie Grosgeorges, Laura Veyrie, Simon Thezenas, Brice Pastor, Ekaterina Pisareva, Gérald Lossaint, Evelyne Lopez-Crapez, Marc Ychou, Alain R. Thierry, Corinne Prévostel, and Philippe Blache

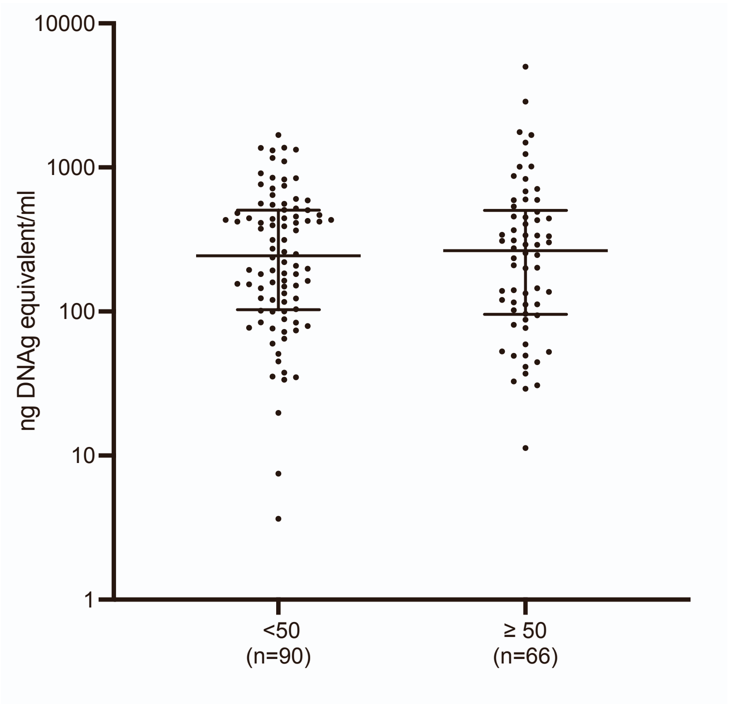

Figure S1: Quantification of TIMP-1 mRNA in healthy women and men aged under 50, 50 or over .

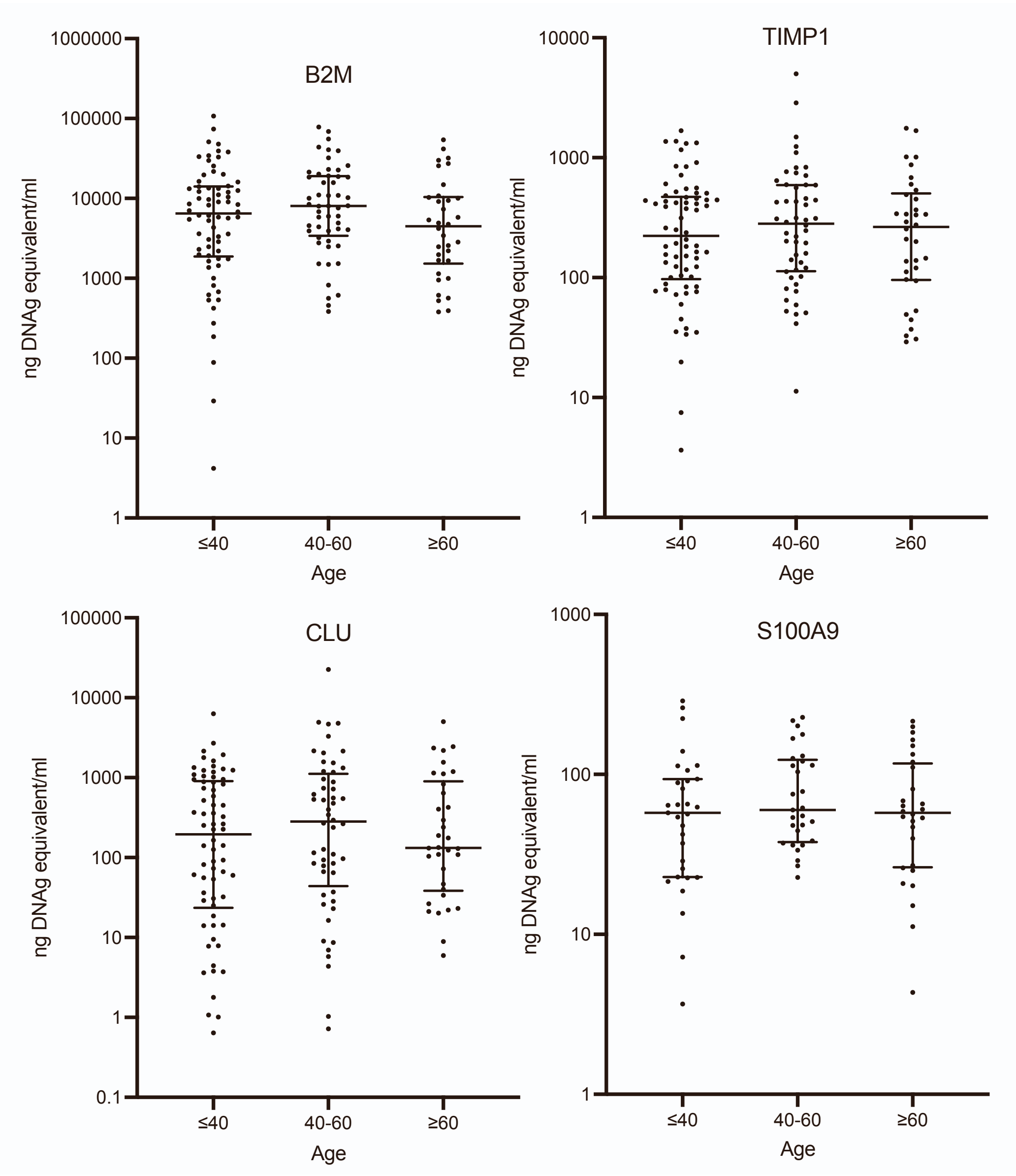

Figure S2:
Analysis of circulating mRNAs (B2M, TIMP1, CLU, and S100A9) in healthy individuals categorized by age group (40 years or younger, 40 to 60 years, and 60 years or older) showing no significant differences between the age groups.

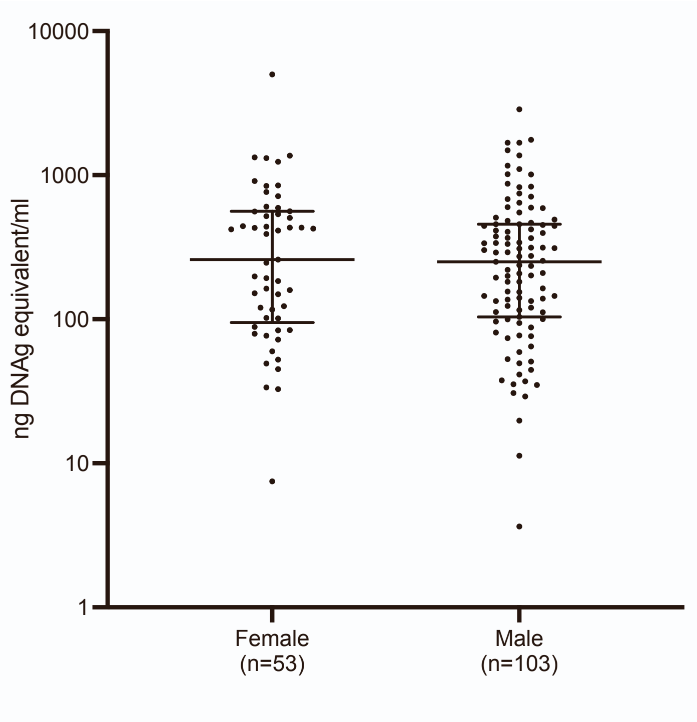

Figure S3: Quantification of TIMP-1 mRNA healthy women versus healthy men.

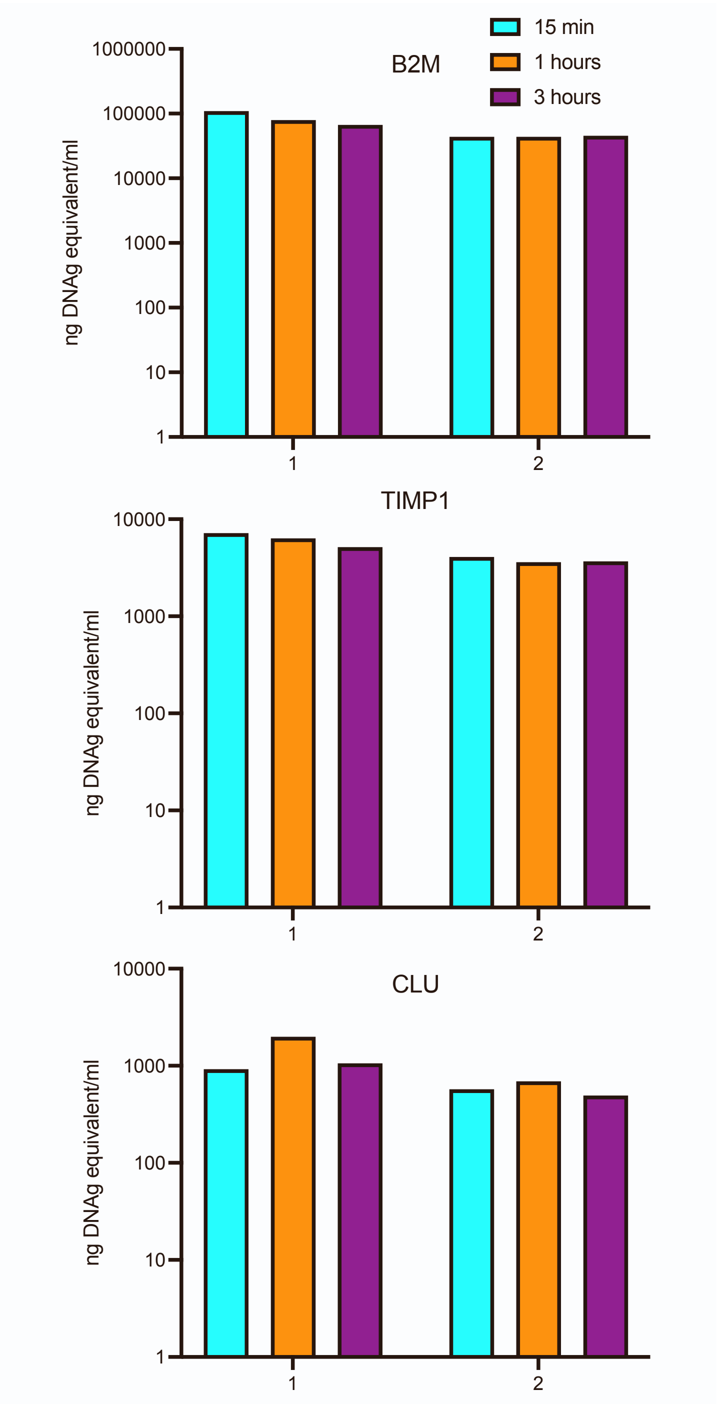

Figure S4: Incidence of the time between blood collection and plasma preparation (15 min, 1 hour or 3 hours) on circulating B2M, TIMP-1 and CLU mRNA analysis (n=2 individuals).

Table Analyzed	MV: Multiple logistic regression (Titanic)			
Dependent variable	mRNA name			
Regression type	Logistic regression			
Model				
Parameter estimates	Variable	Estimate	Standard error	95% CI (profile likelihood)
β0	Intercept	-0.8944	0.9243	-2.782 to 0.8786
β1	B2M	-0.00009168	0.0001745	-0.0004354 to 0.0002493
β2	TIMP-1	0.00852	0.005497	-0.001684 to 0.02013
β3	CLU	-0.003053	0.001623	-0.006203 to ???
β4	B2M^2	0.000000001875	0.000000002462	-2.956e-009 to 6.760e-009
β5	TIMP-1^2	-0.000003282	0.000002059	-7.940e-006 to 1.965e-007
β6	CLU^2	0.000000337	0.0000002952	??? to 8.607e-007
β7	B2M^3	-0.00000000000001125	0.00000000000001463	-4.089e-014 to 1.764e-014
β8	TIMP-1^3	0.0000000004891	0.000000000339	-1.418e-011 to 1.305e-009
β9	CLU^3	-0.00000000001278	0.00000000002308	-4.529e-011 to -1.880e-012
β10	sqrt(B2M)	-0.007588	0.02448	-0.05567 to 0.04031
β11	sqrt(TIMP-1)	-0.1187	0.1474	-0.4193 to 0.1649
β12	sqrt(CLU)	0.1497	0.06038	??? to 0.2697
Odds ratios	Variable	Estimate	95% CI (profile likelihood)	
β0	Intercept	0.4089	0.06190 to 2.407	
β1	B2M	0.9999	0.9996 to 1.000	
β2	TIMP-1	1.009	0.9983 to 1.020	
β3	CLU	0.997	0.9938 to ???	
β4	B2M^2	1	1.000 to 1.000	
β5	TIMP-1^2	1	1.000 to 1.000	

β6	CLU^2	1	??? to 1.000			
β7	B2M^3	1	1.000 to 1.000			
β8	TIMP-1^3	1	1.000 to 1.000			
β9	CLU^3	1	1.000 to 1.000			
β10	sqrt(B2M)	0.9924	0.9458 to 1.041			
β11	sqrt(TIMP-1)	0.8881	0.6575 to 1.179			
β12	sqrt(CLU)	1.162	??? to 1.310			
Model diagnostics	Degrees of Freedom	AICc				
Intercept-only model	348	485.7				
Selected model	336	432.5				
Area under the ROC curve						
Area	0.7624					
Std. Error	0.02523					
95% confidence interval	0.7128 to 0.8117					
P value	<0.0001					
Classification table	Predicted healthy	Predicted cancer	Total	% Correctly classified		
Observed healthy	115	56	171	67.25		
Observed cancer	56	122	178	68.54		
Total	171	178	349	67.91		
Negative predictive power (%)	67.25					
Positive predictive power (%)	68.54					
Classification cutoff	0.5					

Pseudo R squared						
Tjur's R squared	0.2019					
Hypothesis tests	Statistic	P value	Null hypothesis	Reject Null Hypothesis?	P value summary	
Hosmer-Lemeshow	14.47	0.0703	Selected model is correct	No	ns	
Data summary						
Rows in table	349					
Rows skipped (missing data)	0					
Rows analyzed (#observations)	349					
Number of cancer	178					
Number of healthy	171					
Number of parameter estimates	13					
#observations/#parameters	26.8					
# of cancer/#parameters	13.7					
# of healthy/#parameters	13.2					

Table S1: Statistical parameters of the logistic regression combining B2M, TIMP-1 and CLU mRNA values calculated using the Prism 10 for macOS software.

	Pre Surgery (n=33)	Post Surgery (n=33)	HI (n=171)	CRC (n=178)
**B2M**				
Median	8450	13739	7336	10609
Mean	10625	77588	12872	15833
SD	9042	186233	16473	19069
SEM	1574	32419	1260	1429
p value		Pre vs Post 0.008/**	Post vs HI 0.0004/***	Post vs CRC 0.023/*
**TIMP-1**				
Median	508	594	292	633
Mean	648	850	467	1111
SD	612	681	634	1401
SEM	106	118	49	105
p value		Pre vs Post 0.2162/ns	Post vs HI <0.0001/****	Post vs CRC 0.8937/ns
**CLU**				
Median	934	1771	263	923
Mean	1014	2244	858	1567
SD	898	1670	1985	2107
SEM	156	291	152	158
p value		Pre vs Post 0.0006/***	Post vs HI <0.0001/****	Post vs CRC 0.0009/***
**S100A9**				
Median	89	42	62.3	94
Mean	113.5	50.4	86.6	138.6
SD	115.7	26.5	66.8	207.2
SEM	20.1	4.6	6.6	15.5
p value		Pre vs Post 0.0001/***	Post vs HI 0.0103/*	Post vs CRC <0.0001/****

Table S2: B2M, TIMP-1, CLU and S100A9 mRNA levels before and after surgery for 33 patients classified as stage II or III. P values were calculated with the non-parametric Wilcoxon– Mann–Whitney test. Related to figure 5.
